# Lower Trochlear Facet Asymmetry Ratio Is Associated with Objective Patellar Instability in a Retrospective MRI Case-Control Study with Patella Alta-Positive Controls

**DOI:** 10.3390/jcm15155940

**Published:** 2026-07-30

**Authors:** Muhammed Kuru, Nizamettin Koçkara, Mehmet Burak Gökgöz, Volkan Gür, Furkan Yapıcı

**Affiliations:** Department of Orthopedics and Traumatology, Faculty of Medicine, Erzincan Binali Yıldırım University, 24100 Erzincan, Türkiye; m_kuru94@hotmail.com (M.K.); nzmttn@yahoo.com (N.K.); dr.m.burakgokgoz@gmail.com (M.B.G.); drvolkangur@hotmail.com (V.G.)

**Keywords:** patellofemoral instability, trochlear facet asymmetry, trochlear dysplasia, tibial tubercle-trochlear groove distance, axial engagement index, Wiberg classification, magnetic resonance imaging, patella alta

## Abstract

**Background/Objectives:** Because patella alta frequently accompanies objective patellar instability, separating trochlear morphology from patellar height is difficult. We evaluated whether the trochlear facet asymmetry ratio was associated with instability among knees with patella alta. **Methods:** This retrospective case-control MRI study included 123 knees: 43 with objective patellar instability, 43 normal-height controls, and 37 patella alta-positive controls without instability identified by re-screening the eligible control pool. Nine morphologic parameters were measured. The primary analysis compared the instability group with patella alta-positive controls; a supportive analysis used the full cohort. **Results:** The facet asymmetry ratio showed a graded pattern across groups (instability, 42.7 ± 12.4%; patella alta-positive controls, 59.2 ± 10.7%; normal-height controls, 70.3 ± 11.4%; *p* < 0.001). In the primary comparison, it had the highest observed apparent area under the curve (0.855), although its confidence interval overlapped with those of quantitative trochlear depth and tibial tubercle-trochlear groove (TT-TG) distance; the Insall–Salvati ratio showed weaker discrimination (0.679). After adjustment for the Insall–Salvati ratio and age, a lower facet asymmetry ratio remained associated with instability (odds ratio per 1-percentage-point increase, 0.903; 95% confidence interval, 0.854–0.956; *p* < 0.001). This association was attenuated after additional adjustment for quantitative trochlear depth or for tibial tubercle-trochlear groove distance together with Wiberg type. Interobserver reliability was excellent (intraclass correlation coefficient, 0.994). **Conclusions:** In this selected cohort, a lower trochlear facet asymmetry ratio was associated with objective patellar instability after adjustment for patellar height and age. The internally derived 49% threshold is exploratory and requires external validation before clinical use.

## 1. Introduction

Patellofemoral instability is a clinically important disorder with a multifactorial pathogenesis involving osseous morphology, soft-tissue restraints, lower-limb alignment, and dynamic muscular control [1,2,3]. In patients with recurrent or objective patellar instability, identifying the dominant anatomic risk factors is essential, because contemporary treatment strategies are increasingly based on correction of specific pathoanatomic abnormalities rather than on the dislocation event alone [4,5,6,7,8].

The major anatomic risk factors traditionally emphasized in patellofemoral instability include patella alta, trochlear dysplasia, increased tibial tubercle-trochlear groove (TT-TG) distance, and patellar tilt [4,5,6,7]. These parameters are commonly used to guide surgical planning, particularly when medial patellofemoral ligament reconstruction, tibial tubercle osteotomy, distalization, medialization, or trochleoplasty is being considered [8,9,10]. Nevertheless, trochlear morphology is more complex than can be fully captured by a single dysplasia measurement or by a qualitative classification system. The proportional development of the medial and lateral trochlear facets may also influence patellar containment, engagement, and lateral translation during early flexion.

Femoral trochlear facet asymmetry is an imaging parameter that reflects the relationship between the medial and lateral trochlear facets. A reduced medial-to-lateral trochlear facet ratio indicates relative medial facet hypoplasia and greater trochlear asymmetry [11,12]. Although this feature is intuitively relevant for patellar tracking, it has not been examined as extensively as TT-TG distance, patella alta, or conventional quantitative trochlear depths. Contemporary reviews of patellofemoral imaging continue to center on these established descriptors together with the Dejour classification and lateral trochlear inclination [13], and systematic evidence confirms trochlear dysplasia, increased TT-TG distance, and patella alta as the principal morphologic risk factors for first-time and recurrent dislocation [14]. Because these features frequently coexist, the specific contribution of trochlear facet asymmetry is difficult to isolate. In addition, the relationship between trochlear facet asymmetry and patellar morphology remains insufficiently clarified, despite recent evidence suggesting that patellar shape may be associated with femoral trochlear morphology [15].

A particular difficulty is that patella alta accompanies objective instability so frequently that a control group with normal patellar height cannot separate the contribution of trochlear morphology from that of patellar height. The purpose of this study was therefore to evaluate whether a lower trochlear facet asymmetry ratio is associated with objective patellar instability when the comparison group consists of knees that also have patella alta but no objective instability, and to compare its discriminatory performance with established morphologic parameters including TT-TG distance, quantitative trochlear depth, axial engagement index, Wiberg patellar morphology, lateral trochlear inclination, patellar tilt, and the Dejour classification. We hypothesized that a lower facet asymmetry ratio would remain associated with objective patellar instability after accounting for patellar height. Threshold estimation was considered exploratory throughout.

## 2. Materials and Methods

### 2.1. Study Design and Patient Selection

This retrospective case-control study was conducted at a tertiary academic orthopedic center after approval by the Ethics Committee of Erzincan University Faculty of Medicine (approval no. 2023-21/2; date of approval: 30 November 2023). The requirement for informed consent was waived because of the retrospective design and anonymized data analysis. This retrospective case-control study was reported in accordance with the Strengthening the Reporting of Observational Studies in Epidemiology (STROBE) statement; the completed checklist is provided in the Supplementary Materials. Patients who underwent knee imaging between 2018 and 2023 were screened.

The study group consisted of patients with a documented history of patellar dislocation or recurrent objective patellofemoral instability. The morphologic measurements analyzed in this study, including trochlear facet asymmetry ratio, quantitative trochlear depth, TT-TG distance, and axial engagement index, were not used to assign patients to the case group; they were measured after study inclusion for research purposes. This separation between the inclusion criteria and the analyzed morphologic variables was intended to limit incorporation bias. Patients who had undergone surgery for patellofemoral instability were included only if preoperative clinical and imaging records were available. Objective patellar instability was defined as either (i) at least one documented lateral patellar dislocation or (ii) recurrent objective instability documented by the treating surgeon together with a positive apprehension test. Forty cases met the first criterion and three met the second. Patients with subjective instability or anterior knee pain alone, without objective findings, were not included.

**Figure 1:**
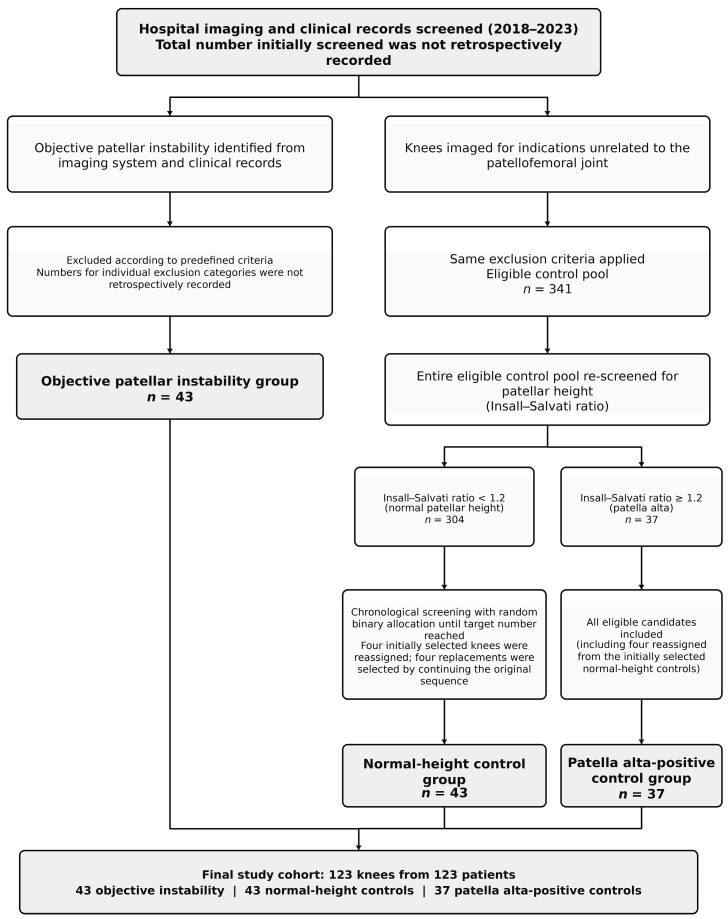
Flow diagram of patient selection. Hospital imaging and clinical records from 2018 to 2023 were screened; the total number initially screened and the numbers corresponding to individual exclusion categories were not retrospectively recorded. Forty-three knees met the criteria for objective patellar instability. The eligible control pool contained 341 knees. Re-screening according to patellar height identified 37 knees with an Insall–Salvati ratio of 1.2 or greater; the remaining 304 knees had an Insall–Salvati ratio below 1.2. The normal-height control group comprised 43 knees selected by chronological screening with random binary allocation, whereas all 37 eligible patella alta-positive controls were included. Four knees initially selected as normal-height controls were reassigned after re-screening and were replaced by continuing the original screening sequence. No individual contributed to more than one group. The final cohort comprised 123 knees from 123 patients.

The objective patellar instability group was identified first from the hospital imaging system. Two separate control groups were then assembled from patients who had undergone knee MRI for indications unrelated to the patellofemoral joint and who had no documented history, symptoms, or clinical findings suggestive of patellar dislocation or recurrent instability. Patients with incomplete imaging, poor-quality MR images, prior major knee surgery affecting patellofemoral anatomy, fracture, tumor, infection, or severe degenerative joint disease were excluded, as were knees with any condition potentially altering trochlear or patellofemoral morphology. The first control group, referred to as the normal-height control group, was drawn from a pool of 341 eligible control candidates listed chronologically by imaging date. Candidates were screened in chronological order, and each was independently assigned a random include-or-exclude outcome until 43 controls had been enrolled to match the size of the case group. Because screening proceeded chronologically and stopped once the target number was reached, earlier candidates had a somewhat higher probability of inclusion than later candidates; this approach therefore approximates, but is not identical to, equal-probability simple random sampling from the full pool. The second control group was constructed specifically to address the near-complete overlap between patella alta and case status observed in the initial analysis. This control group and the associated primary comparison were introduced post hoc during revision in response to concerns regarding confounding by patellar height and were not part of the original analysis plan. Because patella alta is itself an established risk factor for patellar instability, a control group composed almost entirely of knees with normal patellar height does not allow the contribution of trochlear morphology to be separated from that of patellar height. The entire pool of eligible control candidates was therefore re-screened. A lateral radiograph suitable for Insall–Salvati measurement was available for all 341 eligible control candidates, so the re-screening denominator was the complete eligible pool, and every candidate whose lateral radiograph demonstrated an Insall–Salvati ratio of 1.2 or greater was identified. Four such knees had already been enrolled in the normal-height control group and were reassigned; a further 33 were identified among the remaining eligible candidates, giving 37 knees that met the patella alta threshold while having no documented history, symptoms, or clinical findings of objective patellofemoral instability. These knees constituted the patella alta-positive control group. No candidate meeting the threshold was excluded for image quality or any other reason, and no individual contributed to more than one group. Four additional candidates with normal patellar height were enrolled by continuing the original chronological screening sequence from the point at which it had previously stopped, so that the normal-height control group again comprised 43 knees; no separate selection procedure was applied to these four knees. The final study cohort therefore comprised 123 knees from 123 patients: 43 with objective patellar instability, 43 normal-height controls, and 37 patella alta-positive controls. Among the normal-height controls, MRI had been obtained for atraumatic knee pain (*n* = 18), meniscal pathology (*n* = 13), ligamentous evaluation (*n* = 7), and non-patellofemoral cartilage assessment (*n* = 5). Among the patella alta-positive controls, the indications were knee pain (*n* = 17), meniscal pathology (*n* = 11), non-patellofemoral cartilage lesions (*n* = 6), and ligamentous injury (*n* = 3). Normal-height controls were matched to cases in number only and were not individually matched on age, sex, skeletal maturity, or other covariates; the patella alta-positive control group comprised all eligible candidates meeting the patellar height criterion and was therefore not size-matched. The screening and inclusion process is summarized in Figure 1. No a priori sample-size calculation was performed. The study size was determined by all eligible objective-instability cases identified during the study period, a normal-height control group selected to match the number of cases, and all eligible patella alta-positive controls identified by re-screening the complete eligible control pool.

Because controls were selected from clinically indicated MRIs rather than from asymptomatic volunteers, control-group composition was treated as a potential source of selection bias. Available clinical records were reviewed to exclude documented patellofemoral instability, but subclinical patellofemoral symptoms or morphologic variation could not be completely excluded.

### 2.2. Imaging Protocol and Morphologic Measurements

All measurements were performed using knee MRI obtained on a clinical 1.5-T system and lateral knee radiographs available in the institutional picture archiving and communication system. Lateral radiographs permitting identification of patellar length and patellar tendon length for Insall–Salvati measurement were available for all included knees. Because these radiographs had been obtained as part of routine clinical care, knee flexion angle, weight-bearing status, and formal quantitative true-lateral quality criteria were not standardized or consistently retrievable retrospectively. Axial MR images from routine clinical knee MRI protocols were used for trochlear and patellar measurements. All examinations were performed on a single 1.5-T scanner (Magnetom Aera, Siemens Healthineers, Erlangen, Germany) using a dedicated transmit-receive knee coil. The axial proton density-weighted fat-suppressed turbo spin echo sequence used for all trochlear and patellar measurements was acquired with a slice thickness of 3 mm, spacing between slices ranging from 4.05 to 4.65 mm across examinations, a repetition time of 3440 ms, an echo time of 30 ms, an echo train length of 9, two signal averages, an acquisition matrix of 320 × 240, and an in-plane pixel size of 0.56 mm × 0.56 mm. Patients were imaged feet-first in the supine position. Because examinations were obtained as part of routine clinical care rather than under a dedicated research protocol, knee flexion angle and knee rotation were not recorded in the imaging metadata and could not be retrieved retrospectively; these were therefore not entered as covariates and are addressed as a study limitation. Observer 1 measurements were used for the primary group comparison, regression, ROC, and correlation analyses.

**Figure 2:**
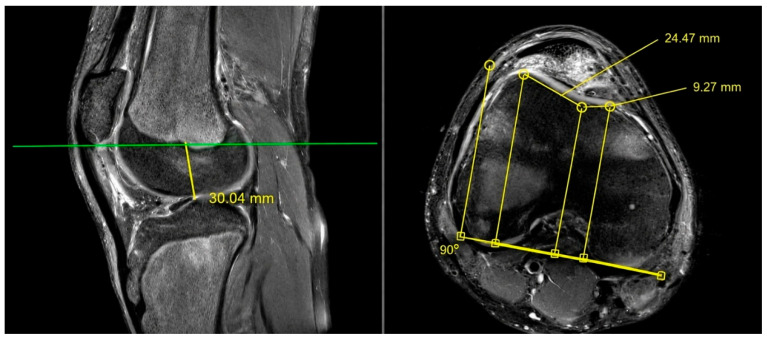
Slice selection and measurement of the trochlear facet asymmetry ratio. The sagittal image demonstrates selection of the axial slice approximately 3 cm proximal to the joint line; the displayed 30.04 mm annotation represents the slice-selection distance and is not used in the facet-ratio calculation. On the axial image, the inferior yellow line represents the posterior condylar tangent. The perpendicular construction lines identify the deepest point of the trochlear groove and the highest bony points of the medial and lateral trochlear facets, while the two superior yellow segments represent the measured medial and lateral facet lengths. The 90° markers indicate perpendicularity to the posterior condylar tangent. The trochlear facet asymmetry ratio is calculated as medial facet length divided by lateral facet length, multiplied by 100.

Trochlear facet asymmetry ratio was measured on a single standardized axial MR slice using bony, rather than cartilaginous, trochlear facet landmarks. The reference slice was defined as the most proximal axial image on which both the medial and lateral bony trochlear facets were fully and simultaneously depicted; when this criterion was met across several adjacent slices, the slice closest to 3 cm proximal to the joint line was selected. The highest point of each facet was defined as the most anterior cortical point of that facet relative to the posterior condylar tangent line (Figure 2). A tangent line was drawn along the posterior femoral condyles. Perpendicular lines were then drawn through the deepest point of the trochlear groove and the highest points of the medial and lateral trochlear facets. Medial and lateral facet lengths were measured, and the trochlear facet asymmetry ratio was calculated as medial facet length divided by lateral facet length, multiplied by 100. Lower values indicated greater medial-to-lateral trochlear facet asymmetry [11,12].

Quantitative trochlear depth was measured at the same axial level using the posterior condylar reference line. It was derived from the perpendicular distances from this reference line to the medial condyle, lateral condyle, and deepest trochlear point. Lower values were interpreted as reflecting a shallower trochlea and thus more pronounced trochlear dysplasia [12,16]. TT-TG distance was measured on axial MRI using the standard posterior condylar reference line, recorded in millimeters as the distance between the perpendicular line through the deepest point of the trochlear groove and the perpendicular line through the center of the tibial tubercle [17,18].

The axial engagement index was measured on axial MRI as the ratio between the trochlear-engaged articular portion of the patella and the total transverse patellar articular width; lower values reflected reduced axial engagement and greater lateral displacement [19]. Patellar morphology was classified on axial MR images according to Wiberg type, with higher types reflecting more pronounced medial facet hypoplasia or convexity [15,20].

Patellar height was assessed in all 123 knees on lateral radiographs using the Insall–Salvati ratio, with a ratio of 1.2 or greater considered consistent with patella alta [21]. The Insall–Salvati ratio was measured in all three groups and was entered as a continuous variable into between-group comparisons, regression analyses, and ROC analyses. Among the normal-height controls, the Insall–Salvati ratio ranged from 0.79 to 1.17; only one knee had a borderline value of 0.79, and all remaining values were greater than 0.80.

For the full cohort, three additional descriptors of trochlear and patellofemoral morphology were measured in all 123 knees. Lateral trochlear inclination was measured on the axial image depicting the proximal trochlea as the angle between the posterior condylar tangent line and a line drawn along the subchondral bone of the lateral trochlear facet, with lower values indicating a flatter lateral facet. Patellar tilt was measured as the angle between the transverse patellar axis and the posterior condylar tangent line, with higher values indicating greater lateral tilt. Trochlear dysplasia was additionally graded using an MRI-adapted application of the Dejour classification (types A to D). Grading was based on axial trochlear morphology, supplemented where necessary by sagittal images, with type A denoting a shallow but symmetrical trochlea, type B a flat or convex trochlea, type C asymmetry between a hypoplastic medial facet and a prominent lateral facet, and type D the additional presence of a cliff pattern between the facets. Because true lateral radiographic criteria such as the crossing sign and the supratrochlear spur could not be assessed uniformly in this retrospective dataset, the grading is described as MRI-adapted rather than as the original radiographic classification. This difficulty is recognized in the literature, and a quantitative MRI-based adaptation of the classification has recently been proposed for precisely this reason [22]. Dejour grading was performed independently by both observers, and interobserver agreement was quantified. Published grouping schemes for the Dejour classification are inconsistent, with low-grade versus high-grade dichotomies variously defined as type A versus types B to D [12,23], types A and C versus types B and D [24], or types A and B versus types C and D. The four categories are therefore reported separately, and the classification was analyzed as an ordinal variable, with a dichotomized analysis (type A versus types B to D) reported as a sensitivity analysis.

### 2.3. Interobserver Reliability

Except for lateral trochlear inclination and patellar tilt, which were measured by a single observer, the radiographic and MRI measurements included in the reliability analysis were independently performed by two orthopedic and traumatology specialists with the same academic rank and equivalent post-specialty experience. Measurements were performed using anonymized images, and the observers were blinded to clinical group allocation and to each other’s measurements. Interobserver reliability for the trochlear facet asymmetry ratio and for the Dejour classification was assessed in all 123 knees, with both observers working independently and blinded to group assignment. For the remaining continuous parameters (TT-TG distance, quantitative trochlear depth, axial engagement index, and Insall–Salvati ratio) and for the Wiberg classification, second-observer measurements were available for the 86-knee reliability subset, and reliability is reported for that subset. Lateral trochlear inclination and patellar tilt were measured by a single observer and are therefore reported as exploratory descriptors without reliability estimates. Continuous parameters were assessed using two-way random-effects, absolute-agreement, single-measure intraclass correlation coefficients [ICC(2,1)], and the ordinal Wiberg and Dejour classifications using quadratic weighted kappa. Bland–Altman bias and 95% limits of agreement were calculated for the continuous parameters. Ninety-five percent confidence intervals were estimated using 5000 non-parametric bootstrap resamples at the image/patient level. Intraobserver reliability was not assessed because repeated same-observer measurements were unavailable; measurement repeatability therefore remains unresolved.

### 2.4. Statistical Analysis

Continuous variables were summarized as mean ± standard deviation and median with interquartile range (IQR) where appropriate, and the ordinal Wiberg classification as median (IQR) with type distribution. Categorical variables were summarized as frequencies and percentages. Normality was assessed using the Shapiro–Wilk test together with inspection of distributions. Independent samples *t*-test or Mann–Whitney U test was used for continuous variables according to distribution. Fisher’s exact test was used for 2 × 2 categorical comparisons.

Comparisons across the three groups were performed using the Kruskal–Wallis test for continuous and ordinal variables and the chi-square test for categorical variables, followed by pairwise Mann–Whitney U tests for the contrasts of interest. To limit the influence of multiple testing across the nine imaging parameters compared between groups, Holm adjustment was applied separately to the nine overall three-group comparisons and to the nine pairwise comparisons for the primary contrast, and adjusted *p* values are reported for both. Univariable logistic regression was performed to evaluate the association between each candidate variable and objective patellar instability. The multivariable analyses were organized into a defined hierarchy. The primary analysis was the comparison between the objective patellar instability group and the patella alta-positive control group, because this contrast directly addresses whether trochlear facet asymmetry is associated with instability among knees that share patella alta status. The primary multivariable model comprised trochlear facet asymmetry ratio, Insall–Salvati ratio, and age. Age was retained in all models because the patella alta-positive control group was older than the other two groups. A supportive full-cohort analysis was then performed in the full cohort of 123 knees using the same model structure, in order to assess the robustness of the primary finding across the wider morphologic spectrum. Separate exploratory models containing the primary model variables together with quantitative trochlear depth, MRI-adapted Dejour grade, TT-TG distance with Wiberg type, or lateral trochlear inclination with patellar tilt were then fitted to examine whether the association between facet asymmetry ratio and instability persisted after adjustment for other descriptors of trochlear morphology. The Insall–Salvati ratio and age were retained in every exploratory model so that adjustment for patellar height was preserved throughout. These models were explanatory and were not intended to define a risk-prediction model. Variance inflation factors were calculated to assess multicollinearity. Because the axial engagement index and the Insall–Salvati ratio are measured on narrow decimal scales, their odds ratios were expressed per 0.1-unit increase. For regression analyses, MRI-adapted Dejour grades A to D were coded from 1 to 4 and entered as an ordinal term, which assumes a linear trend in the log odds across grades. A sensitivity analysis using a dichotomized classification, type A versus types B to D, was therefore also performed. Within the reporting hierarchy, the primary-comparison model comprising the trochlear facet asymmetry ratio, Insall–Salvati ratio, and age was treated as the principal multivariable analysis. The remaining supportive, exploratory, and sensitivity analyses were not treated as confirmatory; no multiplicity adjustment was applied to their model-specific *p* values, which should therefore be interpreted descriptively. Holm adjustment was limited to the overall three-group comparisons and the primary pairwise comparisons across the nine imaging parameters.

ROC curve analysis was used to evaluate apparent within-cohort discriminatory performance, with bootstrap 95% confidence intervals for the area under the curve (AUC) based on 5000 resamples. ROC analyses were performed separately for the primary comparison, for the comparison between cases and normal-height controls, and for the full cohort. Exploratory cut-off values were identified using the Youden index and were not treated as validated clinical thresholds; a threshold was regarded as internally consistent only when it recurred across these analyses. Positive and negative predictive values were not reported because the sampling design does not reflect the prevalence of objective patellar instability in an unselected clinical population. Correlations between trochlear facet asymmetry ratio and the other imaging parameters were assessed using Pearson and Spearman correlation analyses as appropriate. Analyses were performed using SPSS version 27.0 (IBM Corp., Armonk, NY, USA) and R version 4.3.1 (R Foundation for Statistical Computing, Vienna, Austria). A *p* value < 0.05 was considered statistically significant.

Sensitivity analyses comprised restriction to adults aged 18 years or older, an additional restriction to the overlapping age range of 18 to 45 years, exclusion of the three cases included without a radiologically documented dislocation, and use of a dichotomized rather than ordinal Dejour variable. An exploratory within-case comparison was also performed between surgically and non-surgically treated patients. No data were missing for the variables included in the primary and supportive analyses; therefore, no imputation was performed. The availability and scope of second-observer measurements are described in Section 2.3.

## 3. Results

### 3.1. Patient Characteristics

The full cohort comprised 123 knees from 123 patients: 43 with objective patellar instability, 43 normal-height controls, and 37 patella alta-positive controls. Sex distribution and involved side did not differ significantly across the three groups. Height was also comparable. Age differed significantly (*p* < 0.001): patients in the instability group were the youngest (24.6 ± 10.5 years), normal-height controls were intermediate (29.7 ± 12.5 years), and patella alta-positive controls were the oldest (38.1 ± 10.8 years) (Table 1). This gradient reflects the composition of the eligible control pool, which consisted of knee MRIs obtained largely for degenerative and meniscal indications, so that candidates meeting the patella alta criterion were on average older. Age was therefore retained as a covariate in all multivariable models and was additionally addressed in an age-restricted sensitivity analysis. Within the instability group, 40 patients (93.0%) had a documented dislocation and 12 (27.9%) had undergone surgical treatment.

### 3.2. Imaging Parameters Across the Three Groups

All nine imaging parameters differed significantly across the three groups, and all differences remained significant after Holm adjustment for multiple comparisons (Table 2). The Insall–Salvati ratio separated the two control groups by design (0.96 ± 0.10 in normal-height controls versus 1.32 ± 0.12 in patella alta-positive controls) and was highest in the instability group (1.40 ± 0.16). Trochlear facet asymmetry ratio showed a graded pattern in the opposite direction, being lowest in the instability group (42.7 ± 12.4%), intermediate in patella alta-positive controls (59.2 ± 10.7%), and highest in normal-height controls (70.3 ± 11.4%). The difference between the instability group and patella alta-positive controls was highly significant (Holm-adjusted *p* < 0.001), and the two control groups also differed in facet asymmetry ratio. Although both groups met the patella alta threshold, the continuous Insall–Salvati ratio remained slightly higher in the instability group (1.40 versus 1.32; *p* = 0.006); thus, the primary comparison was restricted to patella alta-positive knees but did not constitute matching on continuous patellar height. Quantitative trochlear depth, axial engagement index, and patellar tilt did not differ significantly between the two control groups but differed markedly between the instability group and both control groups. TT-TG distance, lateral trochlear inclination, and Dejour grade differed across all three groups, with patella alta-positive controls occupying an intermediate position.

The distribution of Dejour types followed the same gradient. Type A predominated among normal-height controls (33/43, 76.7%), was less frequent among patella alta-positive controls (15/37, 40.5%), and was uncommon in the instability group (6/43, 14.0%). Conversely, types C and D together accounted for 22 of 43 instability knees (51.2%), 7 of 37 patella alta-positive controls (18.9%), and only 1 of 43 normal-height controls (2.3%) (*p* < 0.001) (Table 3).

### 3.3. Discriminatory Performance in the Primary Comparison

In the primary comparison between the instability group and patella alta-positive controls, trochlear facet asymmetry ratio showed the highest observed apparent AUC among the parameters examined, at 0.855 (95% CI, 0.765–0.932). Quantitative trochlear depth (AUC 0.838) and TT-TG distance (AUC 0.810) followed. The Insall–Salvati ratio, which had almost completely separated cases from normal-height controls in the initial case-versus-normal-height-control analysis, discriminated only weakly when both compared groups met the patella alta threshold (AUC 0.679; 95% CI, 0.556–0.800). The exploratory Youden threshold for facet asymmetry ratio in this comparison was 49%, yielding 81.4% sensitivity (95% CI, 67.4–90.3) and 83.8% specificity (95% CI, 68.9–92.3). The same 49% threshold emerged in the comparison with normal-height controls and in the full cohort (Supplementary Table S1), and this recurrence is reported as an indication of internal consistency rather than as a validated clinical cut-off (Table 4).

### 3.4. Primary Multivariable Analysis

In the primary multivariable model comprising trochlear facet asymmetry ratio, Insall–Salvati ratio, and age, a lower facet asymmetry ratio remained independently associated with objective patellar instability (odds ratio [OR] 0.903 per 1-percentage-point increase; 95% CI, 0.854–0.956; *p* < 0.001), corresponding to an OR of 0.360 per 10-percentage-point increase (95% CI, 0.206–0.638). The Insall–Salvati ratio was not associated with instability in this model (OR 1.454 per 0.1-unit increase; 95% CI, 0.859–2.461; *p* = 0.163), while age showed an inverse association with instability but did not reach statistical significance (OR 0.939 per year; 95% CI, 0.882–1.000; *p* = 0.051). The model AUC was 0.890. In separate exploratory models that retained the Insall–Salvati ratio and age and added other descriptors of trochlear morphology, the association of facet asymmetry ratio with instability persisted after adjustment for MRI-adapted Dejour grade (OR 0.895; 95% CI, 0.833–0.962; *p* = 0.003) and for lateral trochlear inclination with patellar tilt (OR 0.916; 95% CI, 0.857–0.979; *p* = 0.010). The association was attenuated and no longer reached significance when quantitative trochlear depth was included (OR 0.943; 95% CI, 0.881–1.010; *p* = 0.095) or when TT-TG distance and Wiberg type were included together (OR 0.939; 95% CI, 0.881–1.001; *p* = 0.052). In both of these models the Insall–Salvati ratio also remained non-significant, and among the added trochlear parameters only trochlear depth reached significance (Table 5).

### 3.5. Supportive Full-Cohort Analysis

In the supportive analysis of the full cohort of 123 knees, a lower trochlear facet asymmetry ratio remained independently associated with objective patellar instability (OR 0.888 per 1-percentage-point increase; 95% CI, 0.841–0.938; *p* < 0.001), with an apparent model AUC of 0.944. The association persisted across the exploratory full-cohort model specifications, which are reported in Supplementary Table S2. Univariable results for all parameters in both the primary comparison and the full cohort are provided in Supplementary Table S3.

### 3.6. Interobserver Reliability

Interobserver reliability for the trochlear facet asymmetry ratio, assessed in all 123 knees, was excellent (ICC 0.994; 95% CI, 0.991–0.995), with negligible Bland–Altman bias (+0.01%) and limits of agreement of −3.61% to +3.62%. Agreement for the Dejour classification across all 123 knees was high, with a quadratic weighted kappa of 0.916 (95% CI, 0.868–0.951) and exact agreement in 84.6% of knees. For the remaining continuous parameters, assessed in the 86-knee reliability subset, ICC values ranged from 0.987 to 0.992, and agreement for the Wiberg classification was good (weighted kappa 0.867) (Table 6).

### 3.7. Sensitivity Analysis

Restricting the primary comparison to adult patients only, defined as 18 years of age or older, excluded 14 knees, all of which belonged to the instability group, since no patella alta-positive control was younger than 18 years. In the remaining 29 instability knees and 37 patella alta-positive controls, facet asymmetry ratio was lower in the instability group (44.2 ± 13.9% versus 59.2 ± 10.7%; *p* < 0.001; AUC 0.817; 95% CI, 0.695–0.918) and remained associated with instability after adjustment for the Insall–Salvati ratio and age (OR 0.910 per 1-percentage-point increase; 95% CI, 0.862–0.961; *p* < 0.001), while the Insall–Salvati ratio was not associated with instability after adjustment for the facet asymmetry ratio and age (*p* = 0.160). As an additional analysis restricted to the age range in which the two groups overlap, comparison within 18 to 45 years left 27 instability knees and 26 patella alta-positive controls. Facet asymmetry ratio remained significantly lower in the instability group (44.2 ± 13.8% versus 57.1 ± 9.1%; *p* < 0.001; AUC 0.811) and remained associated with instability after adjustment for the Insall–Salvati ratio and age (OR 0.910 per 1-percentage-point increase; 95% CI, 0.856–0.968; *p* = 0.003). Excluding the three cases included without a radiologically documented dislocation did not materially alter the primary comparison (42.2 ± 12.7% versus 59.2 ± 10.7%; *p* < 0.001; AUC 0.857). Replacing the ordinal Dejour variable with a dichotomized low-grade versus high-grade classification also left the association unchanged (OR 0.883; 95% CI, 0.823–0.948; *p* < 0.001). Within the instability group, facet asymmetry ratio did not differ significantly between surgically and non-surgically treated patients (37.4% versus 44.7%; *p* = 0.056). Correlations between facet asymmetry ratio and the other imaging parameters are presented in Supplementary Table S4, and the full sensitivity analysis output is presented in Supplementary Table S5.

## 4. Discussion

The main finding of this study was that a lower femoral trochlear facet asymmetry ratio was associated with objective patellar instability when the primary comparison was restricted to patella alta-positive knees. In this comparison, the facet asymmetry ratio was substantially lower in the instability group and showed the highest observed apparent AUC among the parameters examined, whereas the Insall–Salvati ratio showed weaker discrimination. The facet asymmetry ratio remained associated with instability after adjustment for continuous patellar height and age, although the association was attenuated after adjustment for quantitative trochlear depth or for TT-TG distance together with Wiberg type. These findings support the facet asymmetry ratio as a complementary marker of trochlear morphology within this selected cohort; they do not establish it as a validated diagnostic test, screening tool, or stand-alone clinical decision threshold.

Patellofemoral instability is widely recognized as a multifactorial disorder, and traditional imaging evaluation has focused on patellar height, trochlear dysplasia, TT-TG distance, and patellar tilt [4,5,6,7]. Contemporary reviews continue to organize the imaging assessment of instability around these domains [13], and systematic evidence identifies trochlear dysplasia, increased TT-TG distance, and patella alta as the principal morphologic risk factors for first-time and recurrent dislocation [14]. Because these factors frequently coexist, isolating the contribution of any single parameter is difficult in observational cohorts. The design used here, in which a control group with patella alta but without objective instability was assembled from the same eligible pool, was intended to address precisely this problem for one of the strongest confounders.

The graded pattern observed across the three groups is informative. Facet asymmetry ratio was lowest in the instability group, intermediate in patella alta-positive controls, and highest in normal-height controls, and a similar gradient was seen for Dejour grade, lateral trochlear inclination, and TT-TG distance. This suggests that knees with patella alta but without instability occupy an intermediate morphologic position, carrying some but not all of the features associated with objective instability; recent work likewise indicates that trochlear dysplasia is accompanied by measurable changes in tibial tubercle position, underlining the interdependence of these parameters [25]. Earlier MRI-based topographic work likewise showed that multiple trochlear and patellar measurements differ between control knees and knees with recurrent patellofemoral instability [26]. A relatively hypoplastic medial trochlear facet may represent an altered trochlear architecture that reduces the capacity of the trochlea to engage and guide the patella during early flexion, and the present data indicate that this feature is more pronounced in knees that actually dislocate than in knees that merely share a high-riding patella.

An important question is whether facet asymmetry ratio simply re-expresses information already captured by established descriptors of trochlear dysplasia. In the full cohort, facet asymmetry ratio correlated strongly with Dejour grade, lateral trochlear inclination, patellar tilt, and quantitative trochlear depth, confirming substantial morphologic overlap. Nevertheless, in the primary comparison the association of facet asymmetry ratio with instability persisted after adjustment for Dejour grade and after adjustment for lateral trochlear inclination together with patellar tilt, and in the full cohort it persisted in every model tested, including those containing trochlear depth, Dejour grade, TT-TG distance with Wiberg type, and lateral trochlear inclination with patellar tilt. The association was attenuated in the primary comparison when quantitative trochlear depth was added, and when TT-TG distance and Wiberg type were added together. This attenuation may reflect morphologic overlap, limited statistical power in a subgroup of 80 knees, or both, and the present data cannot distinguish among these explanations; the same models in the larger full cohort retained a significant association. Facet asymmetry ratio is therefore probably best understood as a quantitative descriptor of trochlear morphology that is closely related to, but not fully interchangeable with, existing dysplasia measures.

The relationship between facet asymmetry ratio and the axial engagement index is also morphologically informative. Axial engagement index reflects the transverse relationship between the patella and trochlea and was proposed as an MRI-based method for assessing patellar engagement in instability [19]. A lower facet asymmetry ratio accompanied a lower axial engagement index, suggesting that trochlear asymmetry corresponds to reduced patellar engagement on axial imaging rather than representing an isolated morphologic variant. Similarly, higher Wiberg types were more frequent in the instability group, and the inverse correlation between facet asymmetry ratio and Wiberg type is consistent with the patellofemoral joint developing and functioning as a coupled articulation [15,27]. Whether these relationships reflect developmental coupling, adaptive remodeling, or shared anatomic predisposition cannot be determined from the present data.

The Dejour classification deserves separate comment. Its reproducibility has been repeatedly questioned. In a multicenter reliability study in which 28 experienced examiners graded the same cases, interobserver agreement was only slight, with kappa values of 0.20 on radiographs and 0.13 on MRI, whereas intraobserver agreement was substantially better [28]. Comparable concerns have motivated adapted and quantitative cross-sectional schemes [12], and a quantitative MRI-based version of the classification relying on the sulcus angle, lateral trochlear inclination, and the central trochlear bump has recently been introduced to improve objectivity and repeatability [22]. In the present cohort, agreement between the two observers was high, with a quadratic weighted kappa of 0.916 and exact agreement in 84.6% of knees. This favorable reliability may reflect the use of standardized axial slice selection and the fact that both observers assessed anonymized images using an agreed description. It should nevertheless be interpreted in the context of published evidence indicating that Dejour grading is not uniformly reproducible across readers and centers, and the absence of a consensus low-grade versus high-grade grouping, with schemes variously contrasting type A with types B to D [12,23] or grouping by supratrochlear spur [24], is the reason the four categories are reported separately here.

The threshold of approximately 49% for facet asymmetry ratio recurred in the primary comparison, in the comparison with normal-height controls, and in the full cohort. These analyses were not independent, because they shared the same cases and overlapping control data, so the recurrence reflects internal consistency rather than replication. The threshold was also derived and tested within the same data, and the case-control sampling design tends to inflate apparent discrimination through spectrum effects. The value is therefore reported as an internally consistent exploratory threshold and not as a validated diagnostic cut-off. Predictive values were deliberately not reported, because the proportions of cases and controls were determined by the sampling design rather than by disease prevalence. External validation in independent, consecutively sampled cohorts remains necessary before any threshold could be considered for clinical use.

Measurement technique is an important issue in patellofemoral imaging. Quantitative trochlear measurements may be influenced by axial slice selection, scan orientation, knee rotation, knee flexion, and the posterior condylar reference line [12,16,18,29]. To limit this variability, all examinations were performed on a single scanner using a broadly standardized acquisition protocol, slice selection was standardized to a fixed anatomic level, and interobserver reliability for the facet asymmetry ratio was excellent across all 123 knees, with narrow limits of agreement. Because the facet asymmetry ratio is derived from osseous landmarks, its use may theoretically reduce sensitivity to patellar position compared with measurements that depend on it; this was not directly evaluated in the present study.

This study has several limitations, which can be grouped into design, cohort composition, measurement, and analysis. With respect to design, the study was retrospective, conducted at a single center, and used a case-control structure that does not permit causal inference; controls were patients imaged for other clinical indications rather than asymptomatic volunteers, so subclinical patellofemoral variation cannot be excluded; the absence of objective instability in controls was established from available clinical records rather than from a standardized prospective patellofemoral examination, so some outcome misclassification cannot be excluded. With respect to cohort composition, the patella alta-positive control group was older than the other groups because the eligible pool consisted largely of knees imaged for degenerative and meniscal indications; although age was retained in all models and an age-restricted sensitivity analysis reproduced the primary finding, residual confounding by age cannot be excluded. Because the older age of the patella alta-positive controls was linked to their predominantly degenerative and meniscal imaging indications, age may also represent differences in clinical pathway and potentially in trochlear morphology that cannot be fully removed by covariate adjustment. The normal-height control group was assembled by chronological screening that stopped once the target number was reached and therefore may not fully represent the eligible pool, whereas the patella alta-positive control group comprised every eligible candidate meeting the threshold. Consequently, patella alta was overrepresented in the control spectrum of the supportive full-cohort analysis relative to the eligible source pool, and that analysis should be interpreted as a robustness analysis rather than as a population-representative estimate. Skeletal maturity was not formally assessed using physeal status. With respect to measurement, although observers were blinded to formal group allocation, imaging sequelae of previous patellar dislocation may have been recognizable in some cases, so complete blinding to clinical status cannot be assured. Intraobserver reliability was not assessed, and for parameters other than the facet asymmetry ratio and the Dejour classification, second-observer measurements were limited to the original 86-knee reliability subset; lateral trochlear inclination and patellar tilt were measured by a single observer and are reported as exploratory descriptors; the sulcus angle and the TT-PCL distance could not be assessed reliably in this retrospective dataset and were therefore not analyzed; and although all examinations were acquired on a single scanner using a broadly standardized sequence protocol, knee flexion, knee rotation, and the timing of MRI relative to the dislocation event were not recorded and could not be retrieved retrospectively. With respect to analysis, the exploratory threshold was derived from the same data in which it was tested, the primary comparison included 80 knees and was therefore underpowered for models containing several closely related trochlear parameters, and the multiple exploratory models reported here were explanatory rather than confirmatory.

Despite these limitations, the addition of a patella alta-positive control group addresses the principal confounding problem of the initial case-versus-normal-height-control analysis and strengthens the interpretation that trochlear facet asymmetry is associated with objective patellar instability after accounting for patellar height. At present, the parameter should not be used alone to determine surgical indications; to choose between medial patellofemoral ligament reconstruction, tibial tubercle osteotomy, distalization, medialization, or trochleoplasty; or to define a clinical cut-off. Future studies should validate thresholds in independent consecutively sampled cohorts, assess intraobserver and multicenter reproducibility, compare facet asymmetry directly with the sulcus angle and TT-PCL distance, and determine whether incorporating facet asymmetry into established prediction models for recurrent dislocation [30,31] improves calibration, discrimination, or clinical decision-making.

## 5. Conclusions

Femoral trochlear facet asymmetry, expressed as a reduced medial-to-lateral trochlear facet asymmetry ratio, was associated with objective patellar instability in a retrospective case-control cohort that included patella alta-positive controls without objective instability. In the primary comparison, a lower facet asymmetry ratio remained associated with instability after adjustment for the Insall–Salvati ratio and age. The association persisted in exploratory models containing the MRI-adapted Dejour grade or lateral trochlear inclination with patellar tilt, but was attenuated when quantitative trochlear depth or TT-TG distance together with Wiberg type was included. In the supportive full-cohort analysis, the association remained significant across exploratory model specifications. The facet asymmetry ratio should be regarded as a complementary morphologic marker rather than a validated diagnostic test. The exploratory 49% threshold was derived and evaluated within the same cohort and should not be applied clinically without external validation. Intraobserver and multicenter reliability studies are also required before routine clinical application.

## Figures and Tables

**Table 1:** Baseline characteristics of the three study groups.

Variable	Objective Instability (*n* = 43)	Normal-Height Controls (*n* = 43)	Patella Alta-Positive Controls (*n* = 37)	*p*
Age, years	24.58 ± 10.53	29.67 ± 12.51	38.14 ± 10.77	<0.001
Height, cm	167.79 ± 8.19	169.37 ± 6.38	170.35 ± 5.88	0.147
Female, *n* (%)	27 (62.8)	25 (58.1)	22 (59.5)	0.903
Right knee, *n* (%)	26 (60.5)	17 (39.5)	20 (54.1)	0.139

Values are mean ± standard deviation or *n* (%). *p* values from the Kruskal–Wallis test (age, height) and the chi-square test (sex, side).

**Table 2:** Imaging parameters across the three study groups.

Parameter	Objective Instability (*n* = 43)	Normal-Height Controls (*n* = 43)	Patella Alta-Positive Controls (*n* = 37)	*p* (Holm-Adjusted)	Instability vs. Patella Alta-Positive Controls, Holm-Adjusted *p*
Insall–Salvati ratio	1.40 ± 0.16 1.37 (1.29–1.51)	0.96 ± 0.10 0.95 (0.89–1.04)	1.32 ± 0.12 1.30 (1.25–1.34)	<0.001	0.006
Facet asymmetry ratio, %	42.67 ± 12.40 40.0 (36.3–48.0)	70.28 ± 11.36 70.0 (61.1–79.1)	59.23 ± 10.69 59.3 (52.9–66.0)	<0.001	<0.001
Trochlear depth, mm	3.43 ± 2.18 3.20 (1.73–4.42)	6.21 ± 1.84 6.10 (4.88–7.30)	6.16 ± 1.72 6.15 (4.75–7.45)	<0.001	<0.001
TT-TG distance, mm	15.62 ± 4.46 15.2 (13.62–18.45)	9.66 ± 4.00 9.50 (7.29–11.80)	11.36 ± 2.93 11.5 (9.65–12.70)	<0.001	<0.001
Axial engagement index	0.79 ± 0.18 0.85 (0.69–0.91)	0.94 ± 0.05 0.95 (0.91–0.99)	0.92 ± 0.07 0.92 (0.89–0.99)	<0.001	<0.001
Lateral trochlear inclination, degrees	9.51 ± 7.18 8.66 (4.67–13.13)	19.23 ± 4.00 19.85 (16.25–21.46)	15.62 ± 4.82 16.72 (12.48–18.02)	<0.001	<0.001
Patellar tilt, degrees	21.20 ± 10.10 20.00 (15.88–27.06)	10.00 ± 4.07 9.80 (7.16–13.26)	11.62 ± 7.11 9.53 (7.63–15.55)	<0.001	<0.001
Wiberg type, median (IQR)	2 (2–3)	2 (1–2)	2 (1–2)	<0.001	<0.001
MRI-adapted Dejour grade, median (IQR)	3 (2–3)	1 (1–1)	2 (1–2)	<0.001	0.001

Continuous variables are presented as mean ± standard deviation and median (interquartile range). The two ordinal variables are presented as median (interquartile range). Group comparisons by Kruskal–Wallis test; the final column gives the pairwise Mann–Whitney U *p* value for the primary contrast. Holm adjustment was applied separately across the nine overall comparisons and across the nine pairwise comparisons. No type I patellae were observed in the instability group; the Wiberg type distribution was type II in 25 knees (58.1%) and type III in 18 (41.9%) in this group, type I in 21 (48.8%), type II in 18 (41.9%), and type III in 4 (9.3%) in normal-height controls, and type I in 13 (35.1%), type II in 18 (48.6%), and type III in 6 (16.2%) in patella alta-positive controls. MRI-adapted Dejour grade was analyzed as an ordinal variable (A = 1 to D = 4); the category distribution is given in Table 3. TT-TG, tibial tubercle-trochlear groove.

**Table 3:** Distribution of MRI-adapted Dejour trochlear dysplasia types across the three study groups.

MRI-Adapted Dejour Type	Objective Instability (*n* = 43)	Normal-Height Controls (*n* = 43)	Patella Alta-Positive Controls (*n* = 37)
A, *n* (%)	6 (14.0)	33 (76.7)	15 (40.5)
B, *n* (%)	15 (34.9)	9 (20.9)	15 (40.5)
C, *n* (%)	14 (32.6)	1 (2.3)	6 (16.2)
D, *n* (%)	8 (18.6)	0 (0.0)	1 (2.7)

Overall distribution *p* < 0.001 by the chi-square test; because three expected cell counts were below five, the result was confirmed by a Monte Carlo exact test with 20,000 permutations (*p* < 0.001). The four categories are reported separately because published low-grade versus high-grade groupings of the Dejour classification are inconsistent.

**Table 4:** Discriminatory performance of imaging parameters in the primary comparison (objective instability versus patella alta-positive controls, *n* = 80).

Parameter	AUC	95% CI	Exploratory Cut-Off	Sensitivity, %	Specificity, %
Facet asymmetry ratio	0.855	0.765–0.932	≤49%	81.4 (67.4–90.3)	83.8 (68.9–92.3)
Trochlear depth	0.838	0.745–0.919	≤3.50 mm	58.1	97.3
TT-TG distance	0.810	0.703–0.904	≥13.53 mm	76.7	83.8
Patellar tilt	0.797	0.695–0.893	≥16.07°	74.4	81.1
Lateral trochlear inclination	0.758	0.646–0.860	≤8.88°	55.8	94.6
Axial engagement index	0.747	0.635–0.849	≤0.86	60.5	86.5
Wiberg type	0.730	0.630–0.824	≥2	100.0	35.1
MRI-adapted Dejour grade	0.714	0.604–0.817	≥C	51.2	81.1
Insall–Salvati ratio	0.679	0.556–0.800	≥1.37	53.5	86.5

AUC confidence intervals from 5000 bootstrap resamples. Cut-offs were derived within the same cohort using the Youden index and are exploratory. Predictive values are not reported because the sampling design does not reflect disease prevalence. AUC, area under the curve; CI, confidence interval. Values in parentheses for the facet asymmetry ratio sensitivity and specificity are Wilson 95% confidence intervals.

**Table 5:** Multivariable logistic regression models in the primary comparison (objective instability versus patella alta-positive controls, *n* = 80).

Model and Variables	OR	95% CI	*p*
Primary model			
Facet asymmetry ratio (per 1-percentage-point increase)	0.903	0.854–0.956	<0.001
Insall–Salvati ratio (per 0.1)	1.454	0.859–2.461	0.163
Age (per year)	0.939	0.882–1.000	0.051
Exploratory model adding MRI-adapted Dejour grade			
Facet asymmetry ratio (per 1-percentage-point increase)	0.895	0.833–0.962	0.003
Insall–Salvati ratio (per 0.1)	1.470	0.862–2.506	0.157
Age (per year)	0.939	0.881–1.000	0.050
MRI-adapted Dejour grade (per grade)	0.834	0.369–1.885	0.663
Exploratory model adding LTI and patellar tilt			
Facet asymmetry ratio (per 1-percentage-point increase)	0.916	0.857–0.979	0.010
Insall–Salvati ratio (per 0.1)	1.447	0.852–2.455	0.171
Age (per year)	0.943	0.883–1.008	0.083
Lateral trochlear inclination (per degree)	0.981	0.847–1.135	0.796
Patellar tilt (per degree)	1.020	0.911–1.142	0.727
Exploratory model adding trochlear depth			
Facet asymmetry ratio (per 1-percentage-point increase)	0.943	0.881–1.010	0.095
Insall–Salvati ratio (per 0.1)	1.395	0.788–2.468	0.253
Age (per year)	0.940	0.880–1.004	0.064
Trochlear depth (per mm)	0.677	0.462–0.992	0.046
Exploratory model adding TT-TG and Wiberg type			
Facet asymmetry ratio (per 1-percentage-point increase)	0.939	0.881–1.001	0.052
Insall–Salvati ratio (per 0.1)	1.345	0.808–2.239	0.255
Age (per year)	0.938	0.876–1.003	0.062
TT-TG distance (per mm)	1.167	0.958–1.422	0.124
Wiberg type (per grade)	2.093	0.637–6.881	0.224

All models contain the primary model variables, so the Insall–Salvati ratio and age are retained throughout. Model AUC values were 0.890 (primary model), 0.891 (MRI-adapted Dejour), 0.890 (LTI and tilt), 0.901 (trochlear depth), and 0.906 (TT-TG and Wiberg). Variance inflation factors did not exceed 2.56 in any model. OR, odds ratio; CI, confidence interval; LTI, lateral trochlear inclination; TT-TG, tibial tubercle-trochlear groove.

**Table 6:** Interobserver reliability of imaging measurements.

Parameter	*n*	Statistic	Estimate	95% CI	Limits of Agreement
Facet asymmetry ratio	123	ICC(2,1)	0.994	0.991–0.995	−3.61 to +3.62
MRI-adapted Dejour classification	123	Weighted kappa	0.916	0.868–0.951	Not applicable
Insall–Salvati ratio	86	ICC(2,1)	0.992	0.990–0.995	−0.060 to +0.061
TT-TG distance	86	ICC(2,1)	0.987	0.974–0.994	−1.46 to +1.62
Trochlear depth	86	ICC(2,1)	0.991	0.987–0.994	−0.65 to +0.64
Axial engagement index	86	ICC(2,1)	0.988	0.975–0.993	−0.05 to +0.05
Wiberg classification	86	Weighted kappa	0.867	0.778–0.933	Not applicable

For parameters other than the facet asymmetry ratio and the Dejour classification, second-observer measurements were available for the 86-knee reliability subset. Lateral trochlear inclination and patellar tilt were measured by a single observer and are reported without reliability estimates. ICC, intraclass correlation coefficient; CI, confidence interval; TT-TG, tibial tubercle-trochlear groove.

## Data Availability

The data presented in this study are available on reasonable request from the corresponding author. The data are not publicly available because the underlying clinical and imaging data are subject to institutional ethics approval and applicable privacy and data-protection requirements.

## Supplementary Materials

The following supporting information can be downloaded at: https://www.mdpi.com/article/10.3390/jcm15155940/s1, Supplementary Table S1: Discriminatory performance of imaging parameters in the two secondary comparisons; Supplementary Table S2: Supportive multivariable logistic regression models in the full cohort (*n* = 123); Supplementary Table S3: Univariable logistic regression for objective patellar instability; Supplementary Table S4: Correlations between trochlear facet asymmetry ratio and other imaging parameters in the full cohort (*n* = 123); Supplementary Table S5: Sensitivity analyses for the primary comparison and an exploratory within-case subgroup analysis; Supplementary File: completed STROBE checklist.
