# Supplementary material for: Lower Trochlear Facet Asymmetry Ratio Is Associated with Objective Patellar Instability in a Retrospective MRI Case-Control Study with Patella Alta-Positive Controls
Source: J Clin Med. 2026 Jul 30;15(15):5940. doi: 10.3390/jcm15155940 (PMC13467188; doi:10.3390/jcm15155940)
Supplement: Supplementary file 1 [file jcm-15-05940-s001.zip › Strobe Checklist.pdf]

STROBE Statement: Checklist of items that should be included in reports of case-control studies

Manuscript: Lower Trochlear Facet Asymmetry Ratio Is Associated with Objective Patellar Instability in a Retrospective MRI Case-Control Study with Patella Alta-Positive Controls.

Item	Recommendation	STROBE guideline	Location in the revised manuscript
**Title and abstract**			
**1**	Title and abstract	(a) Indicate the study's design with a commonly used term in the title or the abstract. (b) Provide in the abstract an informative and balanced summary of what was done and what was found.	Title; Abstract
**Introduction**			
**2**	Background/rationale	Explain the scientific background and rationale for the investigation being reported.	Introduction, paragraphs 1-3
**3**	Objectives	State specific objectives, including any prespecified hypotheses.	Introduction, final paragraph
**Methods**			
**4**	Study design	Present key elements of study design early in the paper.	Methods 2.1
**5**	Setting	Describe the setting, locations, and relevant dates, including periods of recruitment, exposure, follow-up, and data collection.	Methods 2.1
**6**	Participants	(a) Give the eligibility criteria, and the sources and methods of case ascertainment and control selection. Give the rationale for the choice of cases and controls. (b) For matched studies, give matching criteria and the number of controls per case.	Methods 2.1; Figure 1. (b) Two control groups were used. Normal-height controls (n = 43) were matched to cases in number only and not individually matched on any covariate. Patella alta-positive controls (n = 37) comprised every eligible candidate meeting the patellar height criterion and were therefore not size-matched.
**7**	Variables	Clearly define all outcomes, exposures, predictors, potential confounders, and effect modifiers. Give diagnostic criteria, if applicable.	Methods 2.1 (outcome definition); Methods 2.2 (imaging variables)
**8**	Data sources/measurement	For each variable of interest, give sources of data and details of methods of assessment (measurement). Describe comparability of assessment methods if there is more than one group.	Methods 2.2; Methods 2.3. All three groups were measured using the same protocols by observers blinded to formal group allocation; however, imaging sequelae of previous dislocation may have made complete blinding to clinical status impossible in some cases, as acknowledged in the Discussion limitations.
**9**	Bias	Describe any efforts to address potential sources of bias.	Methods 2.1 (construction of a patella alta-positive control group to separate trochlear morphology from patellar height); Methods 2.3 (blinded independent measurement); Methods 2.4 (age retained in all models; Holm adjustment); Discussion, limitations paragraph
**10**	Study size	Explain how the study size was arrived at.	Methods 2.1. No a priori sample-size calculation was performed. The study size was determined by all eligible objective-instability cases identified during the study period (n = 43), a normal-height control group selected to match the number of cases (n = 43), and all eligible patella alta-positive controls identified by re-screening the complete eligible control pool (n = 37)
**11**	Quantitative variables	Explain how quantitative variables were handled in the analyses. If applicable, describe which groupings were chosen and why.	Methods 2.2 (Dejour reported as four categories and analysed as an ordinal variable, with the rationale for not dichotomising); Methods 2.4 (odds ratios per 0.1 unit for Insall-Salvati ratio and axial engagement index)
**12**	Statistical methods	(a) Describe all statistical methods, including those used to control for confounding. (b) Describe any methods used to examine subgroups and interactions. (c) Explain how missing data were addressed. (d) If applicable, explain how matching of cases and controls was addressed. (e) Describe any sensitivity analyses.	Methods 2.4. (a) Kruskal-Wallis tests with Holm adjustment and logistic regression models with age retained as a covariate. (b) Age-restricted subgroup analyses. (c) No imputation was performed; measurements were complete for all 123 knees, and the scope of second-observer data is stated in Methods 2.3. (d) Controls were not individually matched, so no

			matched analysis was used; instead, a clearly defined post-revision analysis hierarchy was applied, with one primary comparison between cases and patella alta-positive controls and supportive and exploratory analyses using the full cohort. (e) Sensitivity analyses are reported in Results 3.7 and Supplementary Table S5.
**Results**			
**13**	Participants	(a) Report numbers of individuals at each stage of the study. (b) Give reasons for non-participation at each stage. (c) Consider use of a flow diagram.	Methods 2.1; Results 3.1; Figure 1. Figure 1 reports all available participant-flow numbers, including the eligible control pool of 341 knees, the 304 knees with normal patellar height, the 37 knees with patella alta, the reassignment of four initially selected controls, and the final three study groups. The total number initially screened and the numbers corresponding to individual exclusion categories were not retrospectively recorded and are explicitly identified as unavailable.
**14**	Descriptive data	(a) Give characteristics of study participants and information on exposures and potential confounders. (b) Indicate the number of participants with missing data for each variable of interest.	Results 3.1, Table 1; Results 3.2, Tables 2-3. (b) No missing measurements; second-observer coverage is specified in Methods 2.3 and the Table 6 footnote.
**15**	Outcome data	Report numbers in each exposure category, or summary measures of exposure.	Results 3.2, Tables 2-3
**16**	Main results	(a) Give unadjusted estimates and, if applicable, confounder-adjusted estimates and their precision (e.g., 95% confidence interval). (b) Report category boundaries when continuous variables were categorized. (c) If relevant, translate estimates of relative risk into absolute risk for a meaningful time period.	Results 3.3-3.5, Tables 4-5, and Supplementary Table S2; unadjusted estimates are reported in Supplementary Table S3. (b) Patella alta was defined as an Insall-Salvati ratio of 1.2 or greater (Methods 2.2); the exploratory facet asymmetry threshold is reported in Results 3.3. (c) Not applicable to a case-control design.
**17**	Other analyses	Report other analyses done (e.g., analyses of subgroups and interactions, and sensitivity analyses).	Results 3.6 (interobserver reliability, Table 6); Results 3.7 (sensitivity analyses); Supplementary Tables S1-S5.
**Discussion**			
**18**	Key results	Summarise key results with reference to study objectives.	Discussion, first paragraph
**19**	Limitations	Discuss limitations of the study, taking into account sources of potential bias or imprecision. Discuss both direction and magnitude of any potential bias.	Discussion, limitations paragraph, grouped as design, cohort composition, measurement, and analysis
**20**	Interpretation	Give a cautious overall interpretation of results considering objectives, limitations, multiplicity of analyses, results from similar studies, and other relevant evidence.	Discussion, paragraphs 2-8; Conclusions
**21**	Generalisability	Discuss the generalisability (external validity) of the study results.	Discussion, limitations and final paragraphs; Conclusions
**Other information**			
**22**	Funding	Give the source of funding and the role of the funders for the present study and, if applicable, for the original study on which the present article is based.	Funding statement

Note: An Explanation and Elaboration article discusses each checklist item and gives methodological background and published examples of transparent reporting. The STROBE checklist is best used in conjunction with this article (freely available on the Web sites of PLoS Medicine at http://www.plosmedicine.org/, Annals of Internal Medicine at http://www.annals.org/, and Epidemiology at http://www.epidem.com/). Information on the STROBE Initiative is available at http://www.strobe-statement.org.
