# Supplementary material for: Lower Trochlear Facet Asymmetry Ratio Is Associated with Objective Patellar Instability in a Retrospective MRI Case-Control Study with Patella Alta-Positive Controls
Source: J Clin Med. 2026 Jul 30;15(15):5940. doi: 10.3390/jcm15155940 (PMC13467188; doi:10.3390/jcm15155940)
Supplement: Supplementary file 1 [file jcm-15-05940-s001.zip › Supplementary Table S1.pdf]

Supplementary Table S1. Discriminatory performance of imaging parameters in the two secondary comparisons.

**Parameter**	**AUC**	**95% CI**	**Cut-off**	**Sens., %**	**Spec., %**
Cases versus normal-height controls (n = 86)					
Insall-Salvati ratio	1.000	1.000-1.000	≥1.20	100.0	100.0
Facet asymmetry ratio	0.945	0.887-0.986	≤49%	81.4	100.0
Lateral trochlear inclination	0.866	0.778-0.942	≤13.24°	76.7	97.7
MRI-adapted Dejour grade	0.866	0.788-0.932	≥B	86.0	76.7
Patellar tilt	0.857	0.760-0.940	≥15.68°	76.7	93.0
TT-TG distance	0.841	0.753-0.923	≥13.53 mm	76.7	83.7
Trochlear depth	0.834	0.743-0.917	≤3.60 mm	62.8	95.3
Wiberg type	0.805	0.718-0.880	≥2	100.0	48.8
Axial engagement index	0.803	0.700-0.894	≤0.87	62.8	95.3
Full cohort (n = 123)					
Facet asymmetry ratio	0.903	0.834-0.958	≤49%	81.4	92.5
Insall-Salvati ratio	0.852	0.779-0.912	≥1.29	79.1	75.0

Parameter	AUC	95% CI	Cut-off	Sens., %	Spec., %
Trochlear depth	0.836	0.749-0.913	≤3.60 mm	62.8	93.8
Patellar tilt	0.830	0.740-0.911	≥15.68°	76.7	86.2
TT-TG distance	0.827	0.741-0.904	≥13.53 mm	76.7	83.8
Lateral trochlear inclination	0.816	0.724-0.899	≤13.01°	74.4	86.2
MRI-adapted Dejour grade	0.796	0.711-0.872	≥B	86.0	60.0
Axial engagement index	0.777	0.680-0.866	≤0.86	60.5	92.5
Wiberg type	0.770	0.698-0.837	≥2	100.0	42.5

The perfect separation of the Insall-Salvati ratio in this comparison reflects the normal-height control definition together with the observed distribution of patellar height among cases, and should not be interpreted as independently validated diagnostic performance. Cut-offs are exploratory (Youden index) and were derived within the same data. AUC, area under the curve; CI, confidence interval; TT-TG, tibial tubercle-trochlear groove.
