# Supplementary material for: Lower Trochlear Facet Asymmetry Ratio Is Associated with Objective Patellar Instability in a Retrospective MRI Case-Control Study with Patella Alta-Positive Controls
Source: J Clin Med. 2026 Jul 30;15(15):5940. doi: 10.3390/jcm15155940 (PMC13467188; doi:10.3390/jcm15155940)
Supplement: Supplementary file 1 [file jcm-15-05940-s001.zip › Supplementary Table S2.pdf]

Supplementary Table S2. Supportive multivariable logistic regression models in the full cohort (n = 123).

Model and variables	OR	95% CI	p
Base model			
Facet asymmetry ratio (per 1-percentage-point increase)	0.888	0.841-0.938	<0.001
Insall-Salvati ratio (per 0.1)	2.015	1.289-3.148	0.002
Age (per year)	0.958	0.907-1.013	0.136
Adding trochlear depth			
Facet asymmetry ratio (per 1-percentage-point increase)	0.923	0.866-0.983	0.013
Insall-Salvati ratio (per 0.1)	2.104	1.329-3.330	0.002
Trochlear depth (per mm)	0.695	0.476-1.013	0.058
Age (per year)	0.967	0.915-1.021	0.222
Adding MRI-adapted Dejour grade			
Facet asymmetry ratio (per 1-percentage-point increase)	0.881	0.820-0.946	<0.001
Insall-Salvati ratio (per 0.1)	2.042	1.293-3.227	0.002
MRI-adapted Dejour grade (per grade)	0.857	0.377-1.946	0.712
Age (per year)	0.958	0.906-1.013	0.134
Adding TT-TG distance and Wiberg type			
Facet asymmetry ratio (per 1-percentage-point increase)	0.920	0.866-0.977	0.007
Insall-Salvati ratio (per 0.1)	1.915	1.256-2.922	0.003
TT-TG distance (per mm)	1.184	0.981-1.428	0.079
Wiberg type (per grade)	1.685	0.564-5.035	0.350
Age (per year)	0.961	0.907-1.019	0.182
Adding LTI and patellar tilt			
Facet asymmetry ratio (per 1-percentage-point increase)	0.903	0.846-0.963	0.002
Insall-Salvati ratio (per 0.1)	1.989	1.275-3.103	0.003
Lateral trochlear inclination (per degree)	0.981	0.853-1.129	0.790
Patellar tilt (per degree)	1.025	0.917-1.146	0.664
Age (per year)	0.964	0.909-1.021	0.212

Age was retained in all models and is shown for each model. Model AUC values ranged from 0.944 to 0.951. OR, odds ratio; CI, confidence interval; LTI, lateral trochlear inclination; TT-TG, tibial tubercle-trochlear groove.
