# Supplementary material for: Lower Trochlear Facet Asymmetry Ratio Is Associated with Objective Patellar Instability in a Retrospective MRI Case-Control Study with Patella Alta-Positive Controls
Source: J Clin Med. 2026 Jul 30;15(15):5940. doi: 10.3390/jcm15155940 (PMC13467188; doi:10.3390/jcm15155940)
Supplement: Supplementary file 1 [file jcm-15-05940-s001.zip › Supplementary Table S3.pdf]

Supplementary Table S3. Univariable logistic regression for objective patellar instability.

Variable	Primary comparison (n = 80), OR (95% CI)	p	Full cohort (n = 123), OR (95% CI)	p
Facet asymmetry ratio (per 1-percentage-point increase)	0.888 (0.842-0.936)	<0.001	0.861 (0.817-0.908)	<0.001
Insall-Salvati ratio (per 0.1)	1.592 (1.079-2.347)	0.019	2.273 (1.627-3.174)	<0.001
Trochlear depth (per mm)	0.518 (0.386-0.695)	<0.001	0.477 (0.362-0.628)	<0.001
TT-TG distance (per mm)	1.371 (1.167-1.610)	<0.001	1.407 (1.231-1.608)	<0.001
Axial engagement index (per 0.1)	0.409 (0.244-0.687)	0.001	0.302 (0.180-0.504)	<0.001
Lateral trochlear inclination (per degree)	0.852 (0.781-0.928)	<0.001	0.799 (0.735-0.869)	<0.001
Patellar tilt (per degree)	1.146 (1.069-1.229)	<0.001	1.198 (1.117-1.284)	<0.001
MRI-adapted Dejour grade (per grade)	2.541 (1.453-4.445)	0.001	3.978 (2.338-6.767)	<0.001
Wiberg type (per grade)	5.243 (2.154-12.760)	<0.001	5.797 (2.822-11.909)	<0.001
Age (per year)	0.893 (0.847-0.941)	<0.001	0.934 (0.900-0.969)	<0.001
Female sex	1.151 (0.467-2.835)	0.761	1.185 (0.553-2.538)	0.663

The primary comparison contrasts the objective instability group with patella alta-positive controls. OR, odds ratio; CI, confidence interval; TT-TG, tibial tubercle-trochlear groove.
