# Supplementary material for: Lower Trochlear Facet Asymmetry Ratio Is Associated with Objective Patellar Instability in a Retrospective MRI Case-Control Study with Patella Alta-Positive Controls
Source: J Clin Med. 2026 Jul 30;15(15):5940. doi: 10.3390/jcm15155940 (PMC13467188; doi:10.3390/jcm15155940)
Supplement: Supplementary file 1 [file jcm-15-05940-s001.zip › Supplementary Table S4.pdf]

Supplementary Table S4. Correlations between trochlear facet asymmetry ratio and other imaging parameters in the full cohort (n = 123).

Parameter	Correlation method	Coefficient	p
MRI-adapted Dejour grade	Spearman	−0.753	<0.001
Lateral trochlear inclination	Pearson	+0.705	<0.001
Patellar tilt	Pearson	−0.665	<0.001
Trochlear depth	Pearson	+0.628	<0.001
TT-TG distance	Pearson	−0.623	<0.001
Wiberg type	Spearman	−0.600	<0.001
Axial engagement index	Pearson	+0.540	<0.001
Insall-Salvati ratio	Pearson	−0.521	<0.001

A single, pre-selected coefficient is reported for each parameter. The Spearman rank correlation was used for the two ordinal variables, the MRI-adapted Dejour grade and Wiberg type, and the Pearson correlation was used for the continuous variables. Positive coefficients indicate that a higher facet asymmetry ratio accompanies higher values of the compared parameter. TT-TG, tibial tubercle-trochlear groove.
