# Supplementary material for: Lower Trochlear Facet Asymmetry Ratio Is Associated with Objective Patellar Instability in a Retrospective MRI Case-Control Study with Patella Alta-Positive Controls
Source: J Clin Med. 2026 Jul 30;15(15):5940. doi: 10.3390/jcm15155940 (PMC13467188; doi:10.3390/jcm15155940)
Supplement: Supplementary file 1 [file jcm-15-05940-s001.zip › Supplementary Table S5.pdf]

Supplementary Table S5. Sensitivity analyses for the primary comparison and an exploratory within-case subgroup analysis.

Analysis	Groups compared	Facet asymmetry ratio	p	AUC or adjusted OR
Sensitivity analyses				
Adults aged 18 years or older	29 cases vs 37 controls	44.2 ± 13.9% vs 59.2 ± 10.7%	<0.001	AUC 0.817 (0.695-0.918); adjusted OR 0.910 (0.862-0.961), p < 0.001
Age restricted to the overlapping range of 18 to 45 years	27 cases vs 26 controls	44.2 ± 13.8% vs 57.1 ± 9.1%	<0.001	AUC 0.811; adjusted OR 0.910 (95% CI, 0.856-0.968), p = 0.003
Cases without a radiologically documented dislocation excluded	40 cases vs 37 controls	42.2 ± 12.7% vs 59.2 ± 10.7%	<0.001	AUC 0.857
MRI-adapted Dejour grade entered as a dichotomized rather than an ordinal variable	43 cases vs 37 controls	Not applicable	<0.001	Adjusted OR for facet asymmetry ratio 0.883 (0.823-0.948)
Exploratory within-case subgroup analysis				
Surgically vs non-surgically treated cases	12 vs 31 cases	37.4 ± 11.7% vs 44.7 ± 12.3% (median 36.7, IQR 32.4-43.2 vs median 44.0, IQR 37.7-48.0)	0.056	Not applicable

Values are mean ± standard deviation unless otherwise stated. All sensitivity analyses use the primary comparison between the objective instability group and the patella alta-positive controls. In the adult-only analysis, 14 knees were excluded, all from the instability group, because no patella alta-positive control was younger than 18 years; the adjusted odds ratio for that analysis is from the primary model containing the facet asymmetry ratio, the Insall-Salvati ratio, and age. The dichotomized MRI-adapted Dejour analysis replaced the ordinal Dejour variable in the multivariable model containing the facet asymmetry ratio, the Insall-Salvati ratio, and age, and the odds ratio shown is that of the facet asymmetry ratio per 1-percentage-point increase. The comparison of surgically and non-surgically treated cases is a within-case subgroup analysis and is not a sensitivity analysis of the primary result. AUC, area under the curve; IQR, interquartile range; OR, odds ratio.
